# An Analysis of Pay-for-Performance Schemes and Their Potential Impacts on Health Systems and Outcomes for Patients

**DOI:** 10.1155/2019/8943972

**Published:** 2019-06-19

**Authors:** Kwadwo Kyeremanteng, Raphaëlle Robidoux, Gianni D'Egidio, Shannon M. Fernando, David Neilipovitz

**Affiliations:** ^1^University of Ottawa, 75 Laurier Est, Ottawa, Ontario, Canada K1N 6N5; ^2^The Ottawa Hospital, General Campus, 501 Smyth Road, Ottawa, Canada K1H 8L6; ^3^Queen's University, 99 University Avenue, Kingston, Canada K7L 3N6

## Abstract

Pay-for-performance (P4P) programs have been introduced into the Canadian medical system in the last decades. This paper examines the underlying characteristics of P4P and describes both their advantages and drawbacks. Most P4P programs provide the advantage of rewarding medical acts, thus providing an incentive to take on complex patients. There is a variety of nuanced P4P initiatives, which provide financial incentive according to differing criteria, based on quality measures, incentives, and/or benchmark structures. However, there is no conclusive evidence demonstrating that P4P programs provide better value for money than traditional pay schemes, regardless of particular structural choices. Some evidence has even shown that P4P may be detrimental, especially in disadvantaged and high-risk populations. Additionally, there are a number of ethical and practical concerns that arise with the use of P4P, such as the risk of financial incentives being misused or misinterpreted and patients being refused or referred during treatment. P4P initiatives require careful examination and the creation of solid, evidence-based criteria for evaluation and implementation in Canadian medical systems.

## 1. Introduction

While the overall Canadian population ages, medical therapies are becoming more sophisticated and expensive and result in an extended lifespan for many patients. However, such innovation comes with significant financial cost, and the sustainability of the current healthcare system may be in question. In Ontario, the Provincial Government has made a unilateral decision to cut physician salaries of family physicians and specific subspecialists [1]. When that decision was made in 2012, physician salaries represented 20% of the total healthcare budget [2]. Four years later, despite the decision made by the Provincial Government, physician salaries accounted for 25% of this same budget [3]. Cutting salaries may seem to reduce costs in the short term but may also cause long-term issues, by causing an outflux to higher-paying provinces or countries (such as the United States). It also reduces the ability for a province to recruit top-ranked physicians and may lead to inferior quality of care, resulting in higher morbidity and mortality (and thus higher long-term cost).

Not only is there pressure to reduce costs but there is also pressure to simultaneously improve the quality of care. Both hospitals and health authorities have adopted a patient-centered approach [4]. However, physician remuneration is often based on volume with fee-for-service programs. Therefore, there is no financial incentive for quality improvement. Pay-for-performance (P4P) has been suggested as a possible solution for both cost reduction and quality improvement. P4P is a strategy designed to improve healthcare quality through financial incentive [5]. P4P attempts to improve adherence to best practices by providing financial incentive for health practitioners. It is thought that adherence to clinical guidelines improves quality of care and clinical outcomes. Rightly or wrongly, adherence to best clinical guidelines and practice is not always as high as might be expected [6]. It is unclear whether this is secondary to a lack of resources, a lack of time, or a lack of education. At a national level, a number of related initiatives have been shown to improve knowledge translation, suggesting a potential methodology for increasing compliance with official guidelines [7].

Adherence to best practice, however, is not the only measure of performance in P4P. Outcome measures are also a source of evaluation. As will be discussed later on, a key issue lies in determining appropriate outcome measures for evaluation. P4P approaches have been attempted in several countries, including the United States, the United Kingdom, and Australia [5]. Yet, one of the growing concerns about P4P is that its cost-effectiveness has not, or not yet, been validated. There are certainly costs associated with measuring outcomes, as well as with providing bonuses to healthcare practitioners. In addition to this drawback, P4P has the potential to increase healthcare disparity and reduce quality of care depending on its structure [8]. This paper's aim consequently is to evaluate the pros and cons of P4P and to evaluate its place in Critical Care Medicine. Although this paper primarily focuses on implications for the Canadian healthcare systems, a number of examples are extracted from the United States context, due to the growth of federally funded P4P incentives following the Affordable Care Act. Many European countries, such as the UK, also include P4P programs as part of their medical structure. Physicians living in other industrialized countries may nevertheless find that this paper's conclusions can be easily translated to their own realities, while taking into consideration the contextual nature of P4P programs' implementation and eventual success—or lack thereof.

## 2. Main Text

There are several models for payment in today's healthcare system: salary, case-based payments, capitation, and fee-for-service. Salary is a fixed payment over a specific period of time. Case-based payments are payments within a single episode of care, wherein the amount of services required is not reflected by the physician's earnings. Capitation is a set amount for each enrolled patient assigned to a physician or group of physicians, regardless of whether the patients require assistance. Finally, fee-for-service occurs when the physician is paid for services rendered.

All of these methods have merits but also hindrances. Crucial considerations, when evaluating financial incentives, relate to how such incentives will impact volume of patients seen, physician willingness to evaluate complex patients, cost-effectiveness, and quality of care provided. For example, salaried physicians may provide better quality of care to their patients because they have more time and no incentive to increase their natural speed. Alternatively, they are seeing fewer patients and there is no incentive to see complex patients. This approach is therefore unlikely to improve cost-effectiveness. Case-based payments do provide incentive for physicians to see more patients, but may influence physicians to choose less complex patients in order to increase patient volume. Complex patients may be more likely to be hospitalized and require longer stays in hospital, thus reducing time spent on other cases. Because of this issue, case-based payment is also unlikely to be a cost-effective strategy. In addition, it may also cause discrepancy in the quality of care provided for patients, as relatively healthy individuals may receive enhanced care simply because family physicians and specialists are then more likely to take them on. Complex patients would perhaps be avoided, thus increasing their already higher chances of morbidity and mortality.

Capitation provides incentive for physicians to take on more patients and likely decreases the likelihood of physicians providing unnecessary services, but there are again no incentives to provide care for complex patients. In terms of quality of care, physicians can spend more time with their patients, yet complex patients are once again left vulnerable to morbidity, mortality, and the related long-term costs for society at large. Fee-for-service, however, provides incentive for physicians to see more patients but also to offer more services; thus, seeing complex patients is no longer a deterrent. Fee-for-service may still have reduced cost-effectiveness, as physicians may provide unnecessary services to increase revenues. Another point to consider is that physicians do not have a direct incentive to improve quality of care in fee-for-service models, but patients indirectly become more likely to be seen by a specialist quickly and to successfully request procedures or services in a timely fashion.

In Canadian Critical Care Medicine, physicians are typically paid via fee-for-service and less commonly by salary. The advantage of the fee-for-service model is that critically ill patients are receiving expert care despite the complexity of their illnesses or the time of presentation. For example, a salaried intensivist may be less likely to accept the transfer of a critically ill patient outside of regular work hours, because the physician will obtain the same payment, whether the patient is transferred or not. On the contrary, a fee-for-service intensivist will not only get paid for the services provided but will also receive a premium for addressing the patient's needs during the night or weekend. In other words, the physician is incentivized to ensure that the patient will receive an appropriate level of care, within an appropriate timeframe, if paid by the service. A disadvantage of the fee-for-service model in Critical Care Medicine is that patients that do not require ICU level care may receive it so as to boost physician revenues. For example, a patient that could be managed at a peripheral hospital (or in a step-down unit) may be more likely to be transferred and evaluated at a tertiary ICU because of the financial incentives involved. This would be less likely to happen if the physician were salaried. Overall, a fee-for-service model may sacrifice cost-effectiveness but yield improved quality of care.

As can be seen from such examples, P4P attempts to provide financial incentive for quality improvement and implementation of evidence-based practice, while simultaneously avoiding the negative implications of reimbursement schemes that link payment to volume and complexity [5]. Financial incentives are one of the key elements for changing clinical practice [9]. This is not always the case in a fee-for-service model. For example, if a patient experiences complications such as an abscess after surgery, the surgeon that performs the original surgery will now perform a second surgery to correct the complication. Furthermore, these surgeries can be done after normal working hours or on a weekend, leading to increased compensation. Increased financial reimbursement for primary care physicians taking care of complex patients has been associated with correspondingly improved outcomes in the United States [10], but the risk of ethically misguided incentives is nevertheless present.

The previously outlined concerns of the fee-for-service model have been seemingly considered in the creation of P4P programs. There are three key structures for P4P programs, which have been designed to potentially help avoid counterproductive incentives for medical redundancy. The first is an alternative incentive structure. This can be either reward-based or penalty-based. Reward-based incentives would guarantee bonus payments for clinicians meeting specific performance goals. On the other hand, a penalty-based system would involve withholding reimbursement if specific goals are not met. The second approach is a benchmark structure. This can be either absolute performance, in which incentives are provided when performance exceeds a set threshold, or relative performance, for which incentives are provided if performance exceeds that of others or if there is relative improvement compared to one's past performance. Lastly, there is the quality measures structure. This can be process-based, which means that performance is measured through the incorporation of evidence-based practices. It can also be structure-based, so that performance is based on the implementation of evidence-based healthcare structures. Finally, outcome-based quality measures structure incorporates outcomes, such as morbidity and mortality. P4P can focus its structure towards health authorities, hospitals physician groups, or individual physicians. Ideally, the program will target the provider level that is most likely to improve overall quality, depending on a number of characteristics such as hospital size, target population, and geographical location [5]. Thus, P4P can be complex and layered. The distinguishing characteristics of P4P programs are summarized in Table 1.

One of the concerns regarding P4P is that quality can be difficult to measure. Certainly, there are many existing performance indicators, but performance indicators do not always reflect patient outcomes. For example, a hospital may be concerned with the quality of care surrounding knee replacements, typically measured by the wait time to surgery. However, wait times are typically outside the control of individual surgeons, and if a surgery is successful and improves patient quality of life, the wait time for that surgery may be less indicative of the quality of care than other quality-based outcomes. The incidence of complications (such as thrombosis or infection), for example, may be a more appropriate indicator in this case. If the program chooses to compare itself to other centers (as in an absolute performance structure), it is also important to consider patient variables and risk adjustment. For example, Dr. X may have significantly less postoperative complications compared to Dr. Y; but if one examines Dr. Y's patient population, one may notice that Dr. Y operates on older patients, more diabetic patients, and patients that are noted to be much sicker prior to the operation. In fact, if one adjusts for severity of illness and patient variability, Dr. Y may have less postoperative complications.

Another concern regarding P4P is the efficiency measure. Often, efficiency measures are based on the cost of care per episode. They do not always incorporate quality of care, or even outcomes. Ideally, efficiency measures should take cost into account in order to produce a specified level of quality [11]. For example, if the surgeon has high mortality rates, he or she may be viewed as cost-efficient. Indeed, if patients tend to die one or two days after an operation, cost per episode will be low, as compared to discharging a healthy patient three or four days after an operation. Unfortunately, most efficiency measures do not take into account a quality perspective or even an outcome perspective that would help avoid this type of short-term bias [12].

Some penalty-based P4P programs do provide financial penalties for hospital complications that are viewed as preventable. Unfortunately, there are several complications that may not be completely preventable and yet may still trigger penalties. For example, venous thromboembolism can occur on patients despite appropriate preventive therapy. The more appropriate action would then be to receive a penalty if preventative measures were not put in place, rather than solely evaluate outcomes. Another issue is the fact that many P4P initiatives are based on adherence to guidelines, and those guidelines may be aimed at specific populations whose standards of care may not be generalizable. It is consequently important to remember that guidelines are not prescriptive. In fact, adherence to guidelines may occasionally result in poor patient outcome. For example, inpatients are often started on anticoagulation to prevent venous thromboembolism. Recent guidelines would recommend that almost all patients be put on anticoagulation to prevent venous thromboembolisms. Despite this recommendation, if a patient has suffered a recent intracranial haemorrhage, a physician may be reluctant to put this patient on anticoagulation. With a guidelines adherence program, this action may be penalized. Nevertheless, had a physician elected to put an at-risk patient on anticoagulation, the results may have led to a recurrent intracranial haemorrhage, associated with a high mortality risk. In essence, performance indicators may be arduous to determine and uphold, due to the complex, high-stakes decisions that medical providers must take.

It has been suggested, in fact, that public Canadian P4P initiatives are currently lacking in relevance because they are focused on procedures, as opposed to differentiating performance on multiple levels [13]. It must be noted that private P4P initiatives have been noted to be rare in Canada and are typically a means of diversifying physician income by lowering base rate salaries [13]. Nevertheless, this reported procedural focus has also been observed in an array of P4P programs available outside of Canada, in five other industrialized countries including federally governed and decentralized, as well as publicly, partially privately, and privately funded structures [14]. These results suggest that installing reliable and precise goalposts for P4P performance evaluation is a challenge currently experienced internationally, rather than a uniquely Canadian difficulty.

Research has shown that the impact of P4P is mixed. Most studies have found a small benefit to such programs, but the quality of evidence is heterogeneous. For example, in 2007, Lindenauer et al. published a study looking at a reward-based P4P program aiming to improve public reporting, looking at a population of cardiac patients in 613 American hospitals including a federally funded P4P component, compared to 403 American hospitals which relied on base salary [15]. The researchers concluded that P4P did offer small improvements when joined with a public reporting initiative. In the same year, Glickman et al. published a study on quality improvement, using the reward-based P4P for cardiac patients, while studying 54 American hospitals participating in a federally funded P4P pilot project, compared to 446 control hospitals, and found that the program did not appear to impact quality of care [16]. A few years later, a multicountry meta-analysis of P4P was conducted, yielding similarly contradictory results and ultimately concluding that P4P effects can vary widely, depending on the specific analyzed instance and on a number of contextual factors, such as the relative magnitude of the incentives, how the target goals were measured, the length of the intervention, and the type of medical treatment sought [17]. Due to the wide range of contexts found in the thirty-four studies selected for analysis, the authors do not offer specific recommendations as to which combination of factors may be most effective, as data are still lacking to provide this type of foundational hypothesis [17]. As mentioned previously, the magnitude of the offered financial incentive may be one of those factors influencing outcomes of P4P practices. But how much incentive will result in positive outcomes? It is fair to say that more money is likely to create more change? One study suggested that a 5% increase in capitated physician income might be meaningful enough to influence behaviour [18]; yet environment and context also play a role, independent of raw compensation [17].

Over time, supporting evidence for P4P programs' effectiveness has become progressively more scarce. In a Critical Care Medicine context, in three academic hospitals in Pennsylvania including subspecialty and mixed ICUs, P4P has been shown to increase exclusions based on eligibility yet to have no effect on mortality and adjacent outcomes [19]. However, quality of care may vary depending on patient risk and condition severity, and this is not always taken into account when measuring ICU-based patient outcomes [20]. It is consequently a possibility that some subpopulations may benefit from P4P more than others.

There may indeed be some unintended consequences for implementation of P4P programs. In certain circumstances, P4P may not only fail to improve care but may even decrease quality of care. In a reward-based system, physicians may focus on measures that are associated with bonuses and fail to focus on areas that are not being measured but still may have important implications on clinical outcomes. For instance, in the case of a diabetic patient with chronic obstructive pulmonary disease (COPD), if most of the reward-based program is focused on glycemic control, the physician may be more likely to focus on the diabetes relative to the COPD, when the latter may have a larger impact on the patient's quality of life, morbidity, and mortality.

Furthermore, if one looks at absolute performance programs, physicians that are already performing at a high level will benefit regardless of whether they improve from their baselines. Physicians that are considered low performers may be less likely to change their practice because they will not benefit financially from relative improvement in most P4P models. Alternatively, P4P programs may improve documentation but have no impact on quality of care, depending on how the performance indicators are measured. There may also be a misuse of unnecessary therapies. For example, an unnecessary, but financially rewarded, early antibiotic administration may result in increased misuse of antibiotics and higher resistance over time.

Another major concern with P4P is that it may encourage “patient dumping.” For instance, a reward-based P4P program which analyzes outcomes may lead to complicated or sick patients being avoided by physicians, as these patients' health trajectories may impair statistics. As a result, more complicated patients would remain in the periphery, without the care necessary to improve chances of recovery or even survival. Tertiary centers then maintain their statistics and continue to get their bonuses, while peripheral hospitals and their more complex patients suffer. Another effect one might see is that patients might not receive end-of-life care until the measured time period is complete. For example, considering that some centers measure 28-day mortality, an older patient with multiple complications might be kept alive until the 28-day mark has passed, in order to improve the center's statistics. Overall, quality of care may be sacrificed to ensure goals are being met [5].

In this context, P4P has even been shown to actively *decrease* performance when it comes to higher-risk patients. In a study analyzing the health trajectories of a randomly selected 20% of beneficiaries of Medicare's pay-for-service incentive, practices serving higher-risk patients were found to be disproportionately sanctioned by performance adjustments, suggesting that the nature of the current incentives may effectively discourage clinicians from investing in patients with poorer likely outcomes [21]. This finding relates to the importance of ensuring existing healthcare disparities are not further aggravated by misguided incentive programs. It has also been interpreted by some as a sign that P4P programs must not be pursued. Reacting directly to these concerns within the American healthcare framework, some have written that “[These news]… should be the final nail in the coffin of the current generation of P4P” [22].

In the same line of thought, it has been suggested that, in hospitals serving minority populations and patients of relatively low socioeconomic background, quality standards tend to be lower [23], leading to a risk that P4P programs would penalize those “safety-net” institutions. This may subsequently lead to an additional decrease in quality as physicians relocate elsewhere or alternately choose to focus on visible metrics of quality, while forgoing longer-term and subtler measures such as disease prevention.

It may be that P4P encourages improvement in immediate markers of quality but does not impact longer-term outcomes as obviously, if at all. This seems to hold true across a range of medical domains. As such, in one study analyzing 24 hospitals in the northwest of England, although P4P had been associated with a short-term decrease in mortality, this tendency had not been maintained over a longer period, as compared to hospitals which did not adopt the optional, federally implemented quality incentive program [24]. In an outpatient context in America, patients following an insurer-established, P4P-based, medication-assisted treatment were no less likely to be using drugs at follow-up than their counterparts in standard treatment [25]. Similarly, P4P rewards for medication reviews did lead to a marked increase in such reviews for elderly patients, when implemented in the primary care centers of a Swedish county, but the impact on quality improvement and patient outcome remained unclear and ambiguous over time [26].

Partly as a consequence of such findings, it has been strongly suggested that P4P programs should tailor incentives towards providing quality service to higher-risk populations [27], perhaps especially in a context of private or partially privately funded healthcare, such as the American system, in which there may already be sizeable disparities in terms of socioeconomically-influenced access to quality healthcare. In addition, and in order to ensure a clear understanding of P4P programs' impacts over time, readmission rates are being discussed as a potential marker of interest, in parallel to mortality rates. This may help ensure that a high turnover of still-impaired patients does not artificially boost quality measures. All in all, it is recommended that P4P incentives recognize longer-term measures of health in quality of care, such as a focus on community building and continued social support, especially in vulnerable populations such as veterans [28].

In addition to these ethical considerations, several costs need to be considered for the implementation of P4P programs. At a provider level, there are costs associated with acquiring the staff and technology to abstract data from charts. Such activities usually have economies of scale. Physicians that are part of larger groups will have an easier time initiating such programs. From a government perspective, bonuses can be unexpectedly large, particularly if a program has shown promise, which P4P programs have yet to demonstrate unequivocally. This being said, P4P's purpose is to improve quality of care, while at the same time, decreasing overall healthcare costs and improving efficiency. In doing so, its primary focus remains cost-effectiveness, which may be a particularly appropriate focus of economic analysis, while considering patients' quality of life after they have had contact with the healthcare system [29]. As mentioned previously, however, the data are yet to show, without ambiguity, that P4P programs are indeed effective in any one context [5, 30]. It had originally been suggested that this is partly due to a narrow framework while evaluating efficiency of procedures, such as focusing solely on time-bound measures of cost-effectiveness [31]. However, recent evidence suggests that the opposite may be true, with current indications of mixed short-term effects and ambiguous longer-term impacts.

In this regard, several issues arise, regarding P4P's role in Critical Care Medicine. Certainly, Critical Care Medicine would be a key area in which to improve cost-effectiveness, considering the high costs associated with this type of care. It is estimated that 1% of gross national product and 20% of all hospital costs are related to critical care in the United States alone, with the average ICU admission costing roughly $3000–$5000 per day [32, 33]. Undoubtedly, improved efficiency would be of great benefit. And yet, there is no evidence to suggest P4P will improve outcomes and efficiency in Critical Care Medicine. One of the concerns in Critical Care Medicine is that there are few therapies that have been proven to impact mortality or even quality of care, as ICU populations are extremely heterogeneous. Transplant centers, for examples, may see extremely complex cases, whereas much of the ICU literature is based on a relatively simple patient population. Similarly, cases of layered or repeated medical issues, which are commonly seen in hospitals, tend to be excluded from study participation due to the complexity of their health factor evolution. A lung transplant patient, for example, might be excluded from a ventilator-acquired pneumonia (VAP) study. Immunocompromised patients, who are typically colonized with different microbes, will exhibit a unique pattern of antimicrobial selection. Essentially, it is difficult to classify critically ill patients into specific categories. It is consequently arduous to implement guidelines in complex populations that have rarely been studied.

Often, conditions seen in Critical Care Medicine are syndromic rather than disease states, and it may be difficult to reliably diagnose a clear condition. Due to this variability in patient population, an outcome-based program may be inappropriate in the ICU setting. Another issue is that there are often difficulties inherent in the process of diagnosing certain conditions in Critical Care Medicine. For example, VAP has several definitions and often has a subjective element to its diagnosis.

One additional consideration is that Critical Care Medicine has been moving towards a multidisciplinary approach for several years now. In fact, a team-based approach is associated with improved outcomes [34]. Ultimately, the leader is the physician, but the entire team acts as a guide. The P4P format would not be as valuable if it only rewarded the physician in those circumstances. It would be fairer and more efficient if it rewarded the team, the department, or even the hospital itself. Nevertheless, P4P programs may still have an impact on physician satisfaction, which may in turn influence physician availability. There is, to the authors' knowledge, no evidence suggesting that pay-for-performance impacts physician retention. This being said, in a study comparing the attitudes of American and English physicians under P4P models, American physicians were found to express frustration with the model, due to the impact a small number of noncompliant patients may have on overall figures of physician performance, while English physicians did not express corresponding resentment, as they are able to remove noncompliant patients from official statistics [35]. However, American physicians' attitude towards P4P implementation, as expressed through a bicoastal survey of 53 American medical organizations, all participating in some form of pay-for-performance initiative, is globally positive. As a caveat, it was found that physicians surveyed tended to feel that the impact of the program on quality of care was low to moderate, although they did believe that the program was relevant. Physicians also expressed a wish to be provided with a greater magnitude of financial incentives. These tendencies could be found regardless of physicians' types of practice or demographic characteristics [36].

Altogether, P4P practices tend to vary widely and are often implemented on a large scale, which may lead to differential results depending on type of practice, socioeconomic characteristics, and patient population. Those factors are extremely difficult to capture as part of a multihospital survey and may lead to conflicting or ambiguous results. For the above reasons, it is extremely difficult to argue for the implementation of P4P within Critical Care Medicine without more research, especially as relates to the outcomes of P4P programs, from short- and long-term perspectives. Further research may calibrate its methodology in order to use a fine-grain approach to P4P analysis, unearthing specific contexts which may heighten its relevance and gains.

## 3. Conclusion

In conclusion, P4P may provide financial incentive to improve quality of care and cost-effectiveness to Canadian healthcare institutions, but it may also be detrimental to some subpopulations, at least in some of its currently used forms. The evidence is currently not conclusive for the benefits of P4P, although it can be argued that there is still potential. In Critical Care Medicine, this potential is yet to be evaluated and numerous concerns have been identified. As a whole, P4P might involve the risk of patient dumping, ignoring clinical conditions requiring heightened attention, and aggravating performance gaps between high- and more poorly performing physicians. At this time, there does not appear to be enough evidence to support the implementation of P4P in Critical Care Medicine. More research is consequently warranted in order to shed some light on the topic, so as to optimize the likelihood of increased quality of care for all.

## Figures and Tables

**Table 1:** Key distinguishing characteristics of common P4P structures.

Structures	Incentive	Benchmark	Quality measures
Based on comparison with others	Reward-based	Absolute performance	Outcome-based
Based on standards of care	Penalty-based	N/A	Process-based
Based on past evidence	N/A	Relative performance	Structure-based

## Data Availability

The secondary data used to support the findings of this critical analysis are included within the article as references.
